# Transformation of Ginsenosides by *Lactiplantibacillus plantarum* MB11 Fermentation: Minor Ginsenosides Conversion and Enhancement of Anti-Colorectal Cancer Activity

**DOI:** 10.3390/molecules29010027

**Published:** 2023-12-20

**Authors:** Yunjiao Shen, Yansong Gao, Ge Yang, Zijian Zhao, Yujuan Zhao, Lei Gao, Lei Zhao, Shengyu Li

**Affiliations:** 1 Institute of Agricultural Products Processing Technology, Jilin Academy of Agricultural Sciences (Northeast Agriculture Research Center of China), Changchun 130033, China; syj795520@163.com (Y.S.); gysgerry@126.com (Y.G.); yangge1900@163.com (G.Y.); zhaojaas@163.com (Z.Z.); sunny3173@126.com (Y.Z.); redhuman@126.com (L.G.); 2 School of Pharmaceutical Sciences, Changchun University of Chinese Medicine, Changchun 130117, China; zhaolei@ccucm.edu.cn

**Keywords:** probiotic, *Lactiplantibacillus plantarum*, anticancer, ginsenosides, apoptosis, autophagy

## Abstract

The present study aimed to increase the content of minor ginsenosides and enhance the anti-colorectal cancer activity of ginsenosides via biotransformation by *Lactiplantibacillus plantarum* MB11 screened from fermented foods. A subcutaneous transplantation tumor model of murine colorectal cancer CT26 cells was established in mice to study the anticarcinogenic activities and mechanism of fermented total ginsenosides (FTGs). The results showed that *L. plantarum* MB11 fermentation increased the content of minor ginsenosides and decreased that of major ginsenosides. FTGs reduced the tumor weight and size compared with the model group. Immunofluorescence and TdT-mediated dUTP nick end labeling (TUNEL) analysis showed that FTGs significantly increase the number of caspase-3 cells in tumor tissue and induce cell apoptosis. Mechanically, FTGs activate AMPK/mTOR autophagy pathway and regulate JAK2/STAT3 and Bax/Bcl-2/caspase-3 apoptosis pathway. Overall, fermentation with *L. plantarum* MB11 enhanced minor ginsenosides in total ginsenosides, and FTGs induced subcutaneous transplantation tumor autophagy and apoptosis in mice.

## 1. Introduction

Ginseng (*Panax ginseng* C. A. Mey.) is a traditional Chinese medicinal herb with a history of over 2000 years. Gensenosides are the main active ingredients of ginseng, with good therapeutic effects on many diseases, including cardiovascular diseases, neurological disorders, and metabolic diseases [1]. The pharmacological activities of ginsenosides include improving immune function, antioxidation, anti-aggregation, and anti-inflammatory, anti-stress, and anti-fatigue effects [2]. Several studies have demonstrated that ginsenosides have excellent efficacy and safety in treating lung, gastric, liver, and pancreatic cancers [3]. Recent studies have also confirmed the potential of some ginsenosides as promising compounds for treating colorectal cancer [4]; for example, the treatment of hypoxic colorectal cancer cells with ginsenoside Compound K significantly blocked Nur77-mediated carcinogenic signaling, inhibiting the cancer stem-cell-like cells characteristics and metastasis in vitro and in vivo [5]. Ginsenoside Rh2 significantly inhibited colon cancer cell line proliferation, migration, and invasion, and induced apoptosis and G0/G1 cell cycle arrest [6]. Based on previous findings, the possible mechanisms by which ginsenosides exert anti-tumor effects on CRC mainly include regulating autophagy, apoptosis, proliferation, migration, and angiogenesis [4].

The major ginsenosides, such as Rb1, Rb2, and Rg1, account for more than 80% of the ginsenosides and have poor pharmacological activity and rates of absorption in the human body. Major ginsenosides, such as Rb1, Rb2, and Rg1, can be hydrolyzed by removing the sugar groups at the C-3 and/or C-20 positions, resulting in minor ginsenosides [7]. Compared with the main ginsenosides, the solubility of minor ginsenosides decreased, but the permeability of the cell membrane was increased and the bioavailability was improved [8]. A large number of studies have shown that several minor ginsenosides, such as ginsenosides Rg3, Rh2, F2, and CK, have higher pharmacological activity than major ginsenosides [8]. In addition, some studies have shown that the minor ginsenosides are superior to the major ginsenoside in anticancer treatment [9]. Typically, ginsenosides are administered orally, and the bioavailability of ginsenosides is variable in accordance with the conversion capacity of human gut bacteria. Therefore, increasing the content of minor ginsenosides in ginseng is an effective method to enhance the curative effect of ginseng. Currently, the preparation methods for minor ginsenosides mainly include physical, chemical, and biological transformation techniques [10]. Among them, it was found that placing ginsenosides and sulfur powder in a desiccator and leaving it at 25 °C for 12 h resulted in the production of ginsenoside Rh2 [11]. Meanwhile, treating white ginseng extract with 10% citric acid at 100 °C for 1 h resulted in a 10-fold increase in the concentration of ginsenoside Rg3 [12]. Conversely, due to its high substrate specificity, extremely high catalytic efficiency, mild reaction conditions, and environmental friendliness, biological methods such as microbial or enzymatic methods have been proposed [10]. The microorganisms used for ginsenoside transformation include endophytes, engineered microorganisms, and gut microbiota. Some microorganisms, such as *Penicillium chermesinum*, *Saccharomyces cerevisiae*, and *Bacillus subtilis*, have the robust hydrolytic ability for major ginsenosides [13]. Candida allociferrii JNO301 has been shown to increase the content of ginsenosides such as Rd in red ginseng extracts [14]. Probiotics are a class of live bacteria that are beneficial to human health, especially positively affecting intestinal health, and are more suitable for the fermentation of ginsenosides [15].

The method of obtaining minor ginsenosides through probiotic fermentation has developed rapidly in recent years. Probiotics are live microorganisms beneficial to health when ingested in adequate amounts. The most frequently used probiotic strains include *Lactobacillus* and *Bifidobacterium* [16], a group of microorganisms generally recognized as safe (GRAS) and are widely used in the fields of fermented foods and biomedicine due to their various health-promoting effects, such as regulating the gut microbiota, improving immunity, antioxidation, and anti-tumor effects [17]. Several probiotics, including *L. plantarum*, *L. rhamnosus*, *L. acidophilus*, *L. gasseri, Bifidobacterium bifidum*, and *B. longum*, have been utilized for the biotransformation of ginsenosides [18], such as *L. plantarum* CRNB22, have β-glucosidase activity that can convert ginsenoside Rb1 into Rg3 and Rg5 [19]. Probiotic fermentation significantly increases the content of minor ginsenosides, such as Rh2, F2, Rg3, and CK, and enhances their antioxidant, anti-inflammatory, hypoglycemic, and anti-tumor activities [20]. Some differences in the biotransformation of ginsenosides were reported in relation to the probiotic strains used, and these differences may be caused by the specificity of the strains [21,22]. It is more practical to screen strains with efficient transformation capacity. In addition, it has been found that strains with high glycosidase activity can better convert ginsenosides and improve the conversion rate of minor ginsenosides [23]. As a result, we isolated a strain of *L. plantarum* MB11, chosen for its glycosidase activity through pre-screening. In this study, we fermented total ginsenosides using *L. plantarum* MB11, analyzed the content of minor ginsenosides after fermentation, and investigated the therapeutic effects and mechanisms of fermented total ginsenosides (FTGs) on a mouse model of colorectal cancer.

## 2. Results

### 2.1. Transformation of Ginsenosides by L. plantarum MB11 Fermentation

**Figure 1:**
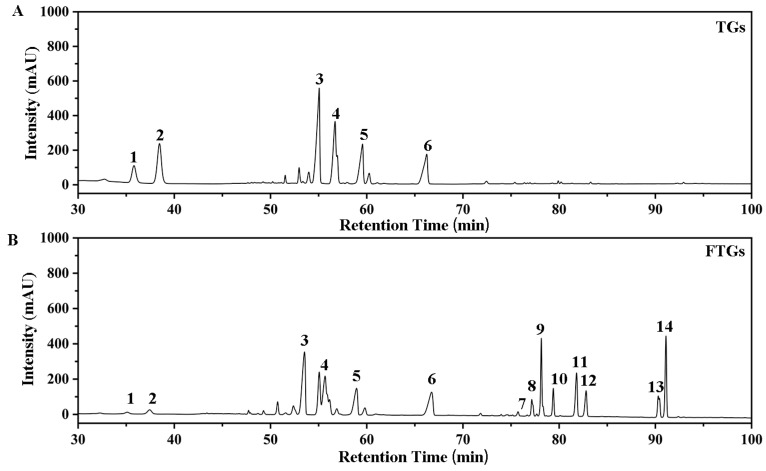
HPLC chromatogram of total ginsenosides fermented with *L. plantarum* MB11 for TGs (**A**) and FTGs (**B**) at 37 °C, 1: Rg1, 2: Re, 3: Rb1, 4: Rb2, 5: Rc, 6: Rd, 7: Rg6, 8: F4, 9: Rk3, 10: Rh4, 11: 20(R)-Rg3, 12: 20(S)-Rg3, 13: CK, 14: Rh2.

The transformation of total ginsenosides (TGs) by *L. plantarum* MB11 was monitored by HPLC. Standard ginsenosides were used as references. As shown in Figure 1A,B, after 21 days of fermentation, the transformation of Rg 1 (peak1), Re (peak2) and Rb 1 (peak3) resulted in eight metabolites (metabolites 7 to 14). The eight metabolites (minor ginsenosides) were identified as Rg6 (peak 7), F4 (peak 8), Rk3 (peak 9), Rh4 (peak 10), Rs3 (peak 11), Rg3 (peak 12), CK (peak 13), and Rh2 (peak 14) by retention time comparison with ginsenoside standards. Ginsenosides Re and Rg1 were hydrolyzed on day 21, where the corresponding peaks (1 and 2) decreased (Figure 1B). Minor ginsenosides were significantly increased in the total ginsenosides fermented by strain MB11. The ginsenoside components of the total ginsenosides fermented by strain MB11 are shown in Table 1. The ginsenoside content of the fermented total ginsenosides (566.33 mg/g) after 21 days was lower than that before fermentation (673.80 mg/g). The total ginsenosides fermented by strain MB11 had the highest content of Rh2 (62.37 mg/g), while the higher content of ginsenoside metabolites Rg6, CK, Rh4, Rk3, and Rg3 was found in the fermented total ginsenosides.

### 2.2. FTGs Inhibit Tumor Growth in CT26-Bearing Mice

**Figure 2:**
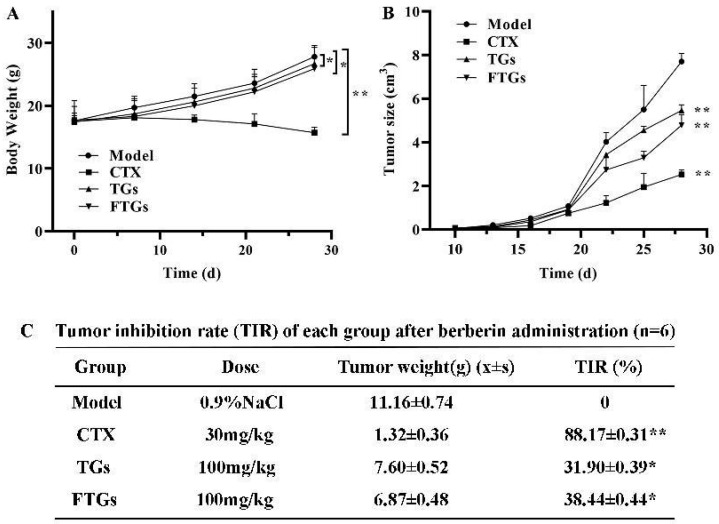
Inhibitory effects of FTGs on the growth of CT26 colon cancer-transplanted tumors in mice. The effects of each group on the body weight (**A**), the size of the tumor (**B**) and the weight of the tumor and the inhibition of the tumor (**C**) in BALB/c mice implanted with CT26 cells. Data are presented as mean ± SD of triplicate experiments, * *p* < 0.05, ** *p* < 0.01 compared with model.

As shown in Figure 2A, FTGs group had lower body weights than model group (*p* < 0.01), with statistically significant differences observed between other groups and model groups (*p* < 0.05). There was a rapid increase in tumor volume in the model group (Figure 2B). In comparison with the model group, the tumor volumes of the TGs and FTGs groups were smaller, and the tumor size of the FTGs was smaller than that of the TGs. As shown in Figure 2C, the tumor inhibition rate was calculated. In terms of tumor weight, both TGs and FTGs treatment significantly reduced tumor weight (*p* < 0.05). The tumor weight of the FTGs group was lighter than that of the TGs group (*p* < 0.05). In addition, the inhibition rate of the FTGs group reached 38.44%, indicating that minor ginsenoside had the best effect with a statistically significant difference.

### 2.3. Effect of FTGs on the Expression of Caspase-3 in Tumor Tissue

**Figure 3:**
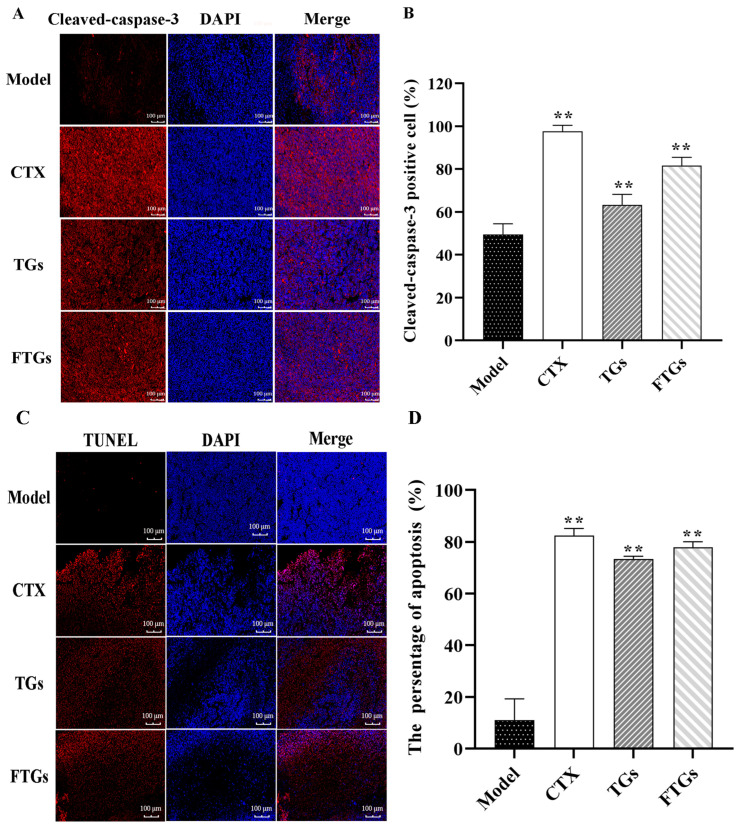
The apoptosis of tumor cells was observed by cleaved-caspase-3 and TUNEL method in FTGs treatment. (**A**) Immunofluorescence staining of cleaved-caspase-3 positive cells in tumor tissue of mice, (**B**) The positive rate of cleaved-caspase-3 cells in each group. (**C**) TUNEL immunofluorescence staining of each group tumor tissue under 20×visual field. (**D**) The percentage of TUNEL positive cells in the tumor tissue of each group. The data were analyzed by one-way ANOVA: ** *p* < 0.01 vs. model group, mean ± SD.

The positive cells rate of cleaved-caspase-3 in the tumor tissue of mice is presented in Figure 3A,B. The number of cleaved-caspase-3 positive cells in tumor tissues was significantly increased in cyclophosphamide (CTX), TGs, and FTGs groups compared with the Model group (Figure 3A, *p* < 0.01). While the rate of cleaved-caspase-3 positive cells was 49.91 ± 3.55% in the Model group, it was 97.35 ± 2.34% in the CTX group and 63.23 ± 3.61% in the TGs group, and 82.44 ± 2.54% in the FTGs group (Figure 3C).

### 2.4. In Vivo Apoptosis Induction by FTGs CT26-Bearing Mice

The TUNEL assay results indicated that the CTX group exhibited the highest positive cell percentage at 82.12%. The apoptosis rate of the FTGs group was higher compared with the TGs group: 75.24% vs. 72.13%. The cell apoptosis was the least in the model group, and the positive cell percentage was 10.54% (Figure 3C,D). Thus, FTGs had a significant anti-tumor effect on CT26-bearing mice via cell apoptosis.

### 2.5. Effect of FTGs on AMPK/mTOR Signaling Pathway in CT26-Bearing Mice

**Figure 4:**
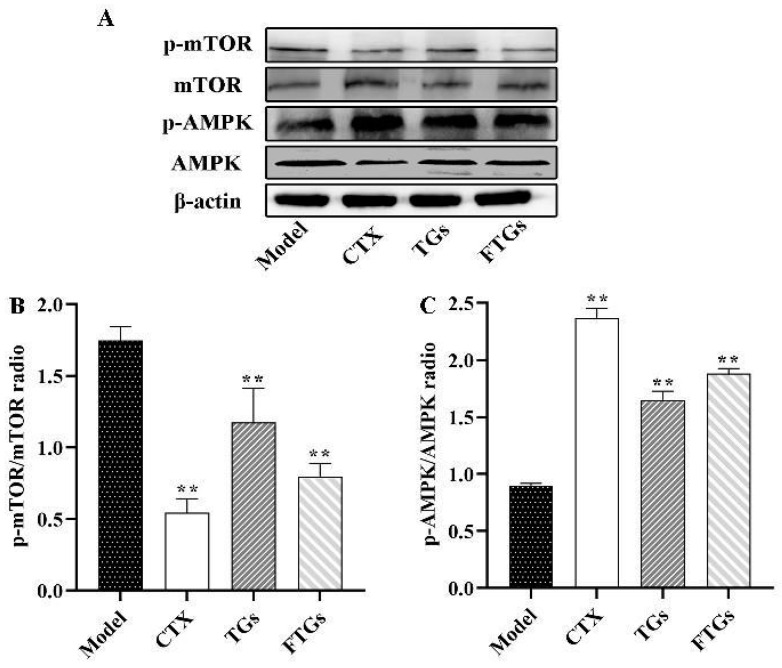
Expressions of phosphorylated and unphosphorylated AMPK and mTOR in CT26-bearing mice (**A**). Representative pictures of western blots (**B**,**C**). Data analyzed by one-way ANOVA: ** *p* < 0.01 vs. model group, mean ± SD. β-actin was used as a standard control for analysis.

In the present study, AMPK and mTOR protein levels were determined to verify the effect of FTGs on CT26-bearing mice through the AMPK/mTOR pathway. As shown in Figure 4, the study found that compared with the treatment group, the model group had higher levels of p-mTOR and lower levels of p-AMPK, but the expression level of p-mTOR proteins was significantly inhibited by FTGs and TGs by 54.60% and 32.18%, respectively (*p* < 0.01). In addition, the expression level of p-AMPK proteins increased significantly compared with that in the model group through FTGs and TGs treatment (1.88 ± 0.04 vs. 0.89 ± 0.02, 1.62 ± 0.03 vs. 0.89 ± 0.02, *p* < 0.05).

### 2.6. FTGs Exert Anticancer Activity via Inhibition of the JAK2/STAT3 Signaling Pathway

**Figure 5:**
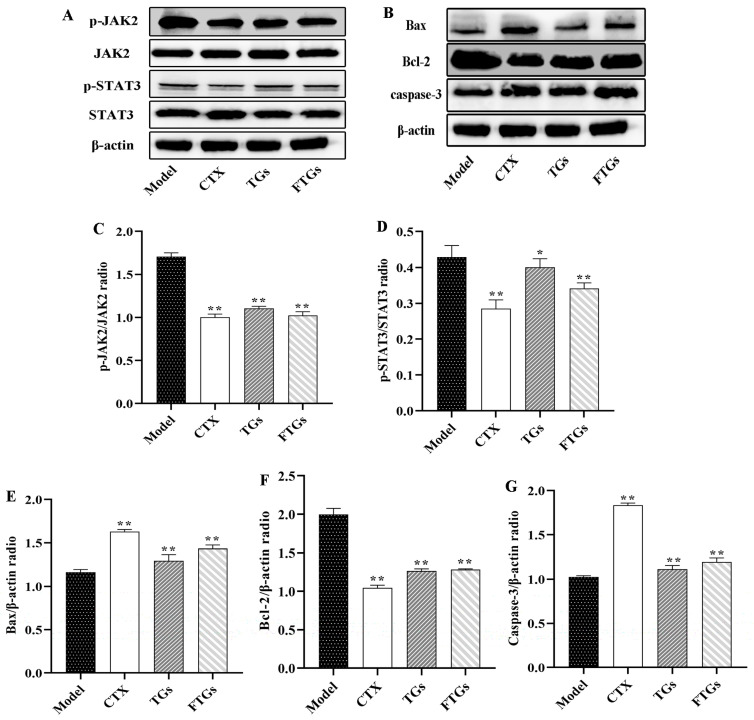
Expressions of phosphorylate JAK2 and STAT3 and unphosphorylated JAK2, STAT3, Bcl-2, Bax and caspase-3 in CT26-bearing mice (**A**,**B**). Representative pictures of western blots (**C**–**G**).One-way ANOVA was used to analyze the data: * *p* < 0.05, ** *p* < 0.01 vs. model group, mean ± SD. β-actin was used as a standard control for analysis.

The expression and phosphorylation of JAK2 and STAT3 in the tumor tissues were detected by Western blotting to investigate the mechanisms underlying FTGs-mediated inhibition of colon tumor growth (Figure 5A). As shown in Figure 5C,D, the overall expression of STAT3 and JAK2 in the tumor tissues was hardly affected. Treatment with FTGs, the expression levels of phosphorylated STAT3 and JAK2 significantly decreased by 42.01% and 18.60%. TGs also significantly reduced the expression levels of phosphorylated STAT3 and JAK2 (*p* < 0.05), with a lower therapeutic effect than FTGs. This may be the critical mechanism underlying the pharmacological properties of FTGs.

### 2.7. Effects of FTGs Treatment on Caspase-3, Bax, and Bcl-2expression in CT26-Bearing Mice

The protein expression of pro and anti-apoptotic markers including Bax, Bcl-2, and caspase-3 was analyzed by western blotting on tumor tissue in CT26-Bearing Mice (Figure 5A). Compared with the Model group, the on CT26-Bearing Mice were treated with TGs and FTGs which significantly reduced the protein expression of Bcl-2 by 35.04% and 40.15% (*p* < 0.05) (Figure 5F). Whereas, those of Bax were increased by 34.61% and 38.34% (Figure 5E, *p* < 0.05), and those of caspase-3 were increased by 35.14% and 40.40% (Figure 5G, *p* < 0.05), respectively, in the TGs- and FTGs- treated CT26 mice. These results indicated that TGs and FTGs could inhibit cell apoptosis, and the effect was better than that of FTGs.

## 3. Discussion

Ginsenosides are the main bioactive compounds in ginseng, according to the difference in the position and quantity of sugar moiety in the glycosides, ginsenosides are divided into three types: protopanaxadiol(PPD), protopanaxatriol (PPT), and oleanolic acid [24]. This study showed a significantly increased content of PPD minor ginsenosides CK, Rh2, 20(R)-Rg3, 20(S)-Rg3 and PPT minor ginsenoside F4, Rh4, Rg6, and Rk3 by fermenting ginsenosides with *L. plantarum* MB11. Consistent with our results, *L. plantarum* M1 and Bacillus subtilis can convert red ginseng as well as ginsenoside Re to the minor ginsenosides Rg3, Rh2, CK, and Rh4, respectively [21,25]. However, recent research has delved into the transformation of major ginsenosides into minor ginsenosides through microbial transformation. This captivating process not only enhances the therapeutic properties of ginsenosides but also opens up new avenues for exploring the symbiotic relationship between these compounds and the microbiome. Ginsenoside Rd and Rg1 were converted to ginsenoside Rd and PPT by *Cellulosimicrobium* sp. TH-20, and it was found that ginsenoside Rd and PPT could dramatically attenuate the production of TNF-α more effectively than the precursors [26]. In addition, microbial and enzyme-conversion techniques are more efficient friendly ways to obtain minor ginsenosides [10]. Markedly, Microorganism mainly act on the sugar moieties of ginsenosides, altering their chemical structures and generating minor ginsenosides that exhibit unique pharmacological properties. For example, *Microbacterium esteraromaticum* can convert ginsenoside Rb1 to CK and its conversion rate can reach 77% [27]. In the study, our results demonstrated that major ginsenoside Rb1 and ginsenoside Re was converted into minor ginsenosides by MB11 with a yield of 87.72% and 88.88%, respectively. Compared with *L. plantarum* M1, *L. plantarum* MB11 has better transformation efficiency [21].

Minor ginsenosides, such as F4, Rg3, Rh4, and CK, have various pharmacological activities, including anti-tumor, anti-fatigue, and neuroprotection [28,29,30,31]. This study showed a significantly increased content of various minor ginsenosides by fermenting ginsenosides with *L. plantarum* MB11. Animal experimental results showed that the anticancer activity of fermented total ginsenosides was enhanced significantly, which might be related to the increase in the content of minor ginsenosides by lactic acid bacteria fermentation [32]. Some studies have shown that ginsenoside Rb1 is transformed into ginsenoside CK by intestinal flora in vivo, significantly improving the therapeutic effect on gastric cancer [33]. Wong et al. also confirmed that ginsenoside Rh2, a secondary saponin degraded after removing glucose from Rg3, has a stronger anticancer activity [34]. The conversion of primary ginsenosides to minor ginsenosides reduces water solubility and increases lipid solubility; minor ginsenosides have a stronger ability to penetrate cell membranes, which might be a major reason for the enhanced activity of minor ginsenosides. In addition, some minor ginsenosides have the same aglycone but different types, numbers, and positions of sugar bases connected; also, their anticancer activities differ significantly. For example, Wan et al. demonstrated that the anti-gastric cancer activity of ginsenoside CK is related to the inhibition of the PI3K/AKT/NF-κB pathway, and the anti-cancer effect of ginsenoside CK is better than that of ginsenoside Rb1 [33]. The above results suggested that extensive screening for the high-efficiency transformation of ginsenosides to prepare minor ginsenosides and lactic acid bacteria strains that can significantly increase the content of ginsenosides with improved anti-tumor activity is essential [35].

Multiple studies have shown that cell-induced autophagy and apoptosis are closely related to cancer treatment, and ginsenosides have been shown to exert an anti-tumor role in apoptosis and autophagy. Among them, the AMPK/mTOR signaling pathway is one of the important pathways that affect the autophagy pathway. Li et al. speculated that ginsenoside CK promotes autophagy-mediated NSCLC cell apoptosis through the AMPK/mTOR and JNK signaling pathways [36]. These findings indicated that the phosphorylation level of AMPK increases and that of mTOR decreases in colon cancer mice treated with FTGs. In addition, JAK2/STAT3 has an anticancer role in many signaling pathways. JAK family proteins are involved in cell differentiation, proliferation, and apoptosis. JAK2 is activated by phosphorylation, which in turn phosphorylates the downstream molecules, including STAT3. Activated p-STAT3 is closely related to apoptosis, autophagy, and the growth of solid tumor cells. The results of this study suggested that FTG treatment can reduce the phosphorylation levels of JAK2 and STAT3. In addition, Zhang colleagues found that ginseng Rh4 inhibited the expression of JAK2, STAT3, and p-STAT3, which inhibited the growth and metastasis of lung cancer [37]. Han et al. also demonstrated that ginsenoside Rh2 inhibits IL-6-induced signal transducer and activator of transcription reduces JAK2 and STAT3 phosphorylation levels and exhibit a role against colorectal cancer [38]. To further elucidate the pro-apoptotic effect of FTGs on colon cancer cells, we detected the expression of Bax, Bcl-2, and caspase-3, which regulates the process of tumor cell apoptosis. The Bcl-2 family of proteins, including Bax and Bcl-2, is the first regulatory step in mitochondrial apoptosis [37]. Bcl-2 is a STAT3-regulated downstream protein that is essential for preventing pro-apoptotic signaling pathways and fostering carcinogenesis, while Bax is the initiator of apoptosis in tumor cells, albeit with reduced levels [39]. The current results indicated that FTGs treatment increases the expression of Bax and decreases the expression of Bcl-2. In addition, cell apoptosis is a gene-regulated programmed cell death process, including caspase-3 activation. The overexpression of caspase-3 is a major executor of tumor cell apoptosis, which can amplify and accelerate apoptosis [40]. In addition, caspase-3 immunofluorescence and TUNEL analyses indicated an increase in cell apoptosis rate. Taken together, these data suggested a pro-apoptotic role of FTGs by inhibiting the STAT3 pathway, upregulating Bax and caspase-3 and downregulating Bcl-2. Moreover, FTGs can antagonize the tumors through the above pathways. In addition, ginsenosides Rg3, CK, etc. can inhibit the proliferation of human colon cancer cells and thus exert anti-tumor effects [41,42]. This also lays the foundation for the subsequent clinical application of FTGs.

## 4. Materials and Methods

### 4.1. Chemicals

Total ginsenosides extracted from ginseng roots purchased from Xi’an Shennong Biotechnology Co., Limited (Xi’an, China) were used as a substrate in this study. Standard ginsenosides, including Rg1, Re, Rb1, Rb2, Rc, Rd, Rg6, F4, Rk3, Rh4, 20(R)-Rg3, 20(S)-Rg3, CK, and Rh2, were obtained from the Chengdu Alfa Biotechnology Co., Limited (Chengdu, China). All other chemicals were reagent grade and obtained from local suppliers.

### 4.2. Lactiplantibacillus Strain and Culture Conditions

β-Glucosidase producing *L*. *plantarum* MB11, isolated from Chinese traditional fermented foods from our laboratory has been used as a starter culture for the production of fermented total ginsenosides. *L. plantarum* MB11 was preserved in the China Center for Type General Microbiological Culture Collection (CCTCC, Wuhan, China) with the accession number CCTCC NO: M2022221. Before use, single colonies were sub-cultured two times in MRS broth, and the activated culture was inoculated at 37 °C for 20 h until its absorbance reached 1 × 10^9^ CFU/mL.

### 4.3. Fermentation of Total Ginsenoside by L. plantarum MB11

The fermentation medium was sterilized at 121 °C for 20 min and consisted of 20 g glucose and 10 g yeast extract per liter. After it has cooled to room temperature, 10% (*w*/*v*) total ginsenosides were added into the fermentation medium after filtration through a 0.22 μm filter. The prepared *L. plantarum* MB11 cells were inoculated into the above fermentation medium for an initial number of viable bacteria at 1.0 × 10^7^ CFU/mL. The inoculated total ginsenosides were then fermented at 37 °C for 21 days. Subsequently, the culture was freeze-dried, dissolved in distilled water, and absorbed on a D101 macroporous resin column for 10 h, followed by gradient elution with water and 30%, 50%, and 70% (*v*/*v*) ethanol. The resin chromatography column was loaded at a flow rate of 0.8 BV/h, and the elution volume of each gradient was 3 BV. The eluted fraction of 50% and 70% ethanol rich in our targeted ginsenosides were vacuum concentrated, were lyophilized to obtain powders, which are then named as FTGs and served as the test samples.

### 4.4. High-Performance Liquid Chromatography (HPLC) Analysis

HPLC analysis was performed as described previously [43]. Briefly, the analysis was carried out on an Agilent HPLC system (Agilent 1260 Infinity, Agilent, Santa Clara, CA, USA) using a pursuit5 SB-C18 column (4.5 mm × 25 cm). HPLC-grade acetonitrile (A) and water (B) were used as mobile phases. The analysis was performed at the flow rate of 1 mL/min, and the gradient elution was as follows: 0~40 min 18~21% (B); 40~42 min 21~26% (B); 42~46 min 26~32% (B); 46~66 min 32~34% (B); 66~71 min 34~38% (B); 71~77.70 min 38.0~49.1% (B); 77.70~82 min 49.1% (B); 82~83 min 49.1~50.6% (B); 83~88 min 50.6~59.6% (B); 88~89.80 min 59.6~65.0% (B); 89.80~97 min 65% (B); 97~102 min 65~75% (B); 102~110 min 75~85% (B); 110~115 min 85% (B); 115~125 min 85~18% (B); 125~130 min 18.0% (B). The injection volume was 20 μL, the column temperature was 30 °C, and the UV absorption was measured at a wavelength of 203 nm. Ginsenoside transformation was monitored by comparing the chromatogram to that of standard ginsenosides. The quantitative data for each ginsenoside were obtained by comparison to known standards.

### 4.5. Cell Line and Cell Culture

The mouse colon cancer cell line CT26 was obtained from Jiangsu KeyGEN BioTech Co., Limited. (Nanjing, China) and maintained in RPMI-1640 medium with 10% fetal bovine serum, 100 IU/mL penicillin, and 100 μg/mL streptomycin. The cell cultures were incubated in a humidified atmosphere of 5% CO_2_ at 37 °C and passaged every 2–3 days. Following the growth and expansion in vitro, the trypsinised cells were harvested, washed and counted by the trypan blue dye exclusion method. After centrifugation, cells were resuspended in phosphate-buffered saline (PBS, pH = 7.2) and seeded at 1 × 10^7^ cells/mL for further syngeneic grafts.

### 4.6. Animal Model and Treatment

A total of 40 BALB/c mice (female, 18–22 g body weight) were purchased from Changchun Yisi Laboratory Animal Technology Co., Limited (Changchun, China) and acclimatized for 1 week before use. All the experimental animal protocols used in this study were carried out in accordance with the standards of the Animal Care and Ethics Committee of Jilin Academy of Agricultural Sciences. An equivalent of 1 × 10^7^ viable tumor cells was suspended in 0.2 mL PBS and inoculated subcutaneously (s.c.) into the right flanks of mice. After the tumors developed to approximately 0.5 cm [44], the mice were randomized into four groups: model group, CTX group, TGs group and FTGs group. The mice were treated with total ginsenosides and *L. plantarum* MB11 fermented total ginsenosides at 100 mg/kg/day in the TGs and FTGs groups. FTGs and TGs were both dissolved in 0.05% Sodium carboxymethyl cellulose (CMC-Na). The CTX group was injected intraperitoneally with CTX at 25 mg/kg/day, and the model group was given 0.9% NaCl by gavage.

During administration, vital signs and food consumption were observed daily, body weight and tumor size were measured and recorded every 7 and 3 days, respectively, and tumor volume was calculated using the following equation: tumor volume = (length × width^2^) × 1/2. After the final treatment, the mice were sacrificed to collect the tumors, which were dissected and weighed. Then, the tumor inhibition index (TIR) was calculated as 100% × (mean tumor weight of the control group or mean tumor weight of the treatment group)/mean tumor weight of the control group. The tumors were rapidly frozen in liquid nitrogen and stored at −80 °C for further analysis.

### 4.7. Immunofluorescence Staining of Caspase-3

The fixed tumor tissue was embedded in paraffin and sliced into 20 μm coronal sections. Then, the slices were deparaffinized with xylene and ethanol, sealed with PBS containing 0.5% Triton X-100, blocked with 5% bovine serum albumin (BSA) for 30 min, and incubated with the cleaved-caspase-3 antibody (1:3000) at 4 °C for 12 h. Subsequently, the slices were incubated with the secondary antibody (1:200) at room temperature for 1 h and developed with 3-diaminobenzidine for 10 min. The number of cleaved-caspase-3 positive cells in the tumors was analyzed using Image J (National Institutes of Health, Bethesda, MD, USA).

### 4.8. TUNEL Assay

Paraffin-embedded sections of tumor tissue from each group were dewaxed in xylene, hydrated with a graded concentrations of ethanol, washed with PBS, dried and incubated with protease K for 20 min at room temperature and rinsed in PBS. A TUNEL working solution (TdT enzyme:dUTP:buffer = 1:5:50) was dripped onto the section, followed by coverage with a preservative film and incubation at 37 °C in the dark for 2 h. Subsequently, the slides were rinsed with PBS, dried, and treated with a DAPI solution containing nucleotides. The apoptotic cells were observed, and images were acquired by fluorescence microscopy. The nuclei were stained blue by DAPI under the excitation of ultraviolet light, and the positive apoptotic nuclei were red.

### 4.9. Western Blot Analysis

Total protein was extracted from tumor tissues. Each tissue sample was homogenized in RIPA lysis buffer with phosphorylase and protease inhibitors (Solarbio, Beijing, China). The tissue supernatant was collected by centrifuging (12,000× *g*, 4 °C) for 10 min. The protein content was determined by BCA kit (UElandy, Suzhou, China) and adjusted to uniform concentration for subsequent experiments. Protein samples were resolved on 8%, 10.0% or 12% SDS-PAGE by boiling with denaturation buffer (1.5 mol Tris-HCl, 10% sodium dodecyl sulfate (SDS), TEMED and 10% ammonium persulphate). The samples were transferred to a Polyvinylidene fluoride (PVDF) (Millipore, MA, USA) for 40 min. A total of 3% BSA in Tris-buffered saline with 0.05% Tween-20 (TBST) (Coolaber, Beijing, China) was used to block the membrane, which was subsequently probed with primary antibodies at 4 °C for 12 h, including rabbit β-actin antibody (GeneTex, GTX629630, Hsinchu, China), rabbit STAT3 antibody (GeneTex, GTX636400, Hsinchu, China), rabbit JAK2 antibody (Bioss, bs-0908R, Beijing, China), rabbit p-STAT3 antibody (Bioss, bs-22386R, Beijing, China), rabbit p-JAK2 antibody (GeneTex, GTX132784, Hsinchu, China), rabbit mTOR antibody (Bioss, bsm-54471R, Beijing, China), rabbit p-mTOR antibody (Bioss, bs-3494R, Beijing, China), rabbit AMPK antibody (Bioss, bs-10344R, Beijing, China), rabbit p-AMPK antibody (Cell Signaling, #2535, Shanghai, China), rabbit Bcl-2 antibody (GeneTex, GTX100064, Hsinchu, China), rabbit caspase-3 antibody (Abcam, ab184787, Cambridge, MA, USA), and rabbit Bax antibody (GeneTex, GTX109683, Hsinchu, China), and then incubated with horseradish peroxidase-conjugated secondary antibody at 37 °C for 1 h. Immunoreactive bands were quantified using Image Quant LAS 4000 (Fuji Film, Tokyo, Japan) with β-actin used as a loading control.

### 4.10. Statistical Analysis

All data are expressed as the mean value ± standard deviation (SD). Experimental results were analyzed for significant differences using one-way ANOVA with Tukey’s multiple comparison. Data were also collated, and bar and line chart constructed using Origin 8.0 software. To indicate a statistically significant difference, *p* < 0.05 was used.

## 5. Conclusions

In conclusion, this study demonstrated that fermentation of ginsenosides by *L. plantarum* increases the content of minor ginsenosides. Nevertheless, exploring different fermentation strains or optimizing fermentation conditions deserves further research in the future. In addition, FTGs activated the AMPK/mTOR autophagy pathway and downregulated the expression of JAK2/STAT3 apoptotic pathway proteins, while upregulated the expression of Bax and caspase-3 proteins and downregulated the expression of Bcl-2 proteins to exert an anti-colon cancer role. Thus, FTGs could be used as natural nutritional supplements in the treatment of colorectal cancer. Though the data presented suggest various mechanisms for the anti-cancer effect of FTGs, the mode of action of FTGs in colon cancer calls for further study and in vivo observations are needed to evaluate the clinical utility of FTGs in colorectal cancer chemoprevention.

## Figures and Tables

**Table 1:** Identification of constituents on HPLC content determination.

Peak	Retention Time (min)	Ginsenoside	TGs Content (mg/g)	FTGs Content (mg/g)
1	35.81	Rg1	77.87 ± 0.23	9.56 ± 0.36
2	38.46	Re	135.74 ± 0.31	15.10 ± 0.42
3	55.06	Rb1	238.15 ± 0.45	161.50 ± 0.74
4	56.71	Rb2	103.43 ± 0.25	74.21 ± 0.52
5	59.56	Rc	35.86 ± 0.54	27.32 ± 0.49
6	66.24	Rd	80.63 ± 0.68	66.72 ± 0.47
7	76.34	Rg6	0	2.56 ± 0.16
8	77.84	F4	0.52 ± 0.12	5.51 ± 0.23
9	78.86	Rk3	0.50 ± 0.11	17.89 ± 0.53
10	80.08	Rh4	0	9.08 ± 0.14
11	82.47	20(R)-Rg3	0	61.82 ± 0.26
12	83.42	20(S)-Rg3	0	37.96 ± 0.48
13	90.08	CK	0	14.73 ± 0.38
14	92.90	Rh2	1.1 ± 0.05	62.37 ± 0.61

## Data Availability

All data were included within this article.
